# PCR-Based Simple Subgrouping Is Validated for Classification of Gliomas and Defines Negative Prognostic Copy Number Aberrations in *IDH* Mutant Gliomas

**DOI:** 10.1371/journal.pone.0142750

**Published:** 2015-11-11

**Authors:** Shunsuke Nakae, Hikaru Sasaki, Saeko Hayashi, Natsuki Hattori, Masanobu Kumon, Yuya Nishiyama, Kazuhide Adachi, Shinya Nagahisa, Takuro Hayashi, Joji Inamasu, Masato Abe, Mitsuhiro Hasegawa, Yuichi Hirose

**Affiliations:** 1 Department of Neurosurgery, Fujita Health University, Toyoake, Aichi, Japan; 2 Department of Neurosurgery, Keio University, Tokyo, Japan; 3 Department of Pathology, Fujita Health University, Toyoake, Aichi, Japan; University Hospital of Navarra, SPAIN

## Abstract

Genetic subgrouping of gliomas has been emphasized recently, particularly after the finding of *isocitrate dehydrogenase 1* (*IDH1*) mutations. In a previous study, we investigated whole-chromosome copy number aberrations (CNAs) of gliomas and have described genetic subgrouping based on CNAs and *IDH1* mutations. Subsequently, we classified gliomas using simple polymerase chain reaction (PCR)-based methods to improve the availability of genetic subgrouping. We selected *IDH1/2* and *TP53* as markers and analyzed 237 adult supratentorial gliomas using Sanger sequencing. Using these markers, we classified gliomas into three subgroups that were strongly associated with patient prognoses. These included *IDH* mutant gliomas without *TP53* mutations, *IDH* mutant gliomas with *TP53* mutations, and *IDH* wild-type gliomas. *IDH* mutant gliomas without *TP53* mutations, which mostly corresponded to gliomas carrying 1p19q co-deletions, showed lower recurrence rates than the other 2 groups. In the other high-recurrence groups, the median progression-free survival (PFS) and overall survival (OS) of patients with *IDH* mutant gliomas with *TP53* mutations were significantly longer than those of patients with *IDH* wild-type gliomas. Notably, most *IDH* mutant gliomas with *TP53* mutations had at least one of the CNAs +7q, +8q, −9p, and −11p. Moreover, *IDH* mutant gliomas with at least one of these CNAs had a significantly worse prognosis than did other *IDH* mutant gliomas. PCR-based mutation analyses of *IDH* and *TP53* were sufficient for simple genetic diagnosis of glioma that were strongly associated with prognosis of patients and enabled us to detect negative CNAs in *IDH* mutant gliomas.

## Introduction

Gliomas are currently classified according to their histological appearance, and the associated malignancy is defined by the World Health Organization (WHO) grading system. In cases of high grade gliomas, patients tend to show high recurrence rates and a worse prognosis. However, in some cases, the clinical course does not reflect the histological classification, warranting the use of genetic diagnoses and subgroups. We previously reported that adult supratentorial gliomas could be classified into genetic subgroups on the basis of their copy number aberrations (CNAs) using comparative genomic hybridization (CGH) and suggested that gliomas with +7q and 1p/19q co-deletions may have a better prognosis than those with −9p, −10q, and +7 CNAs [1].

The clinical significance of *isocitrate dehydrogenase 1* (*IDH1*) point mutation in gliomas was first reported in 2008, the overall survival (OS) of glioblastoma patients with *IDH1*-mutated glioblastoma was demonstrated to be significantly longer than that of patients with wild-type *IDH1* glioblastoma [2]. Various subsequent studies confirmed the prognostic importance of *IDH1* mutations [3–6]. Therefore, we combined a CGH analysis with the *IDH1* mutation status to propose the genetic subgrouping of gliomas [5]. The data demonstrated that *IDH1* mutant gliomas with −1p/19q and +7q CNAs are associated with a better prognosis than that associated with *IDH1* wild-type gliomas.

Although these genetic subgroups were clinically informative, copy number-independent and simplified methods are desirable for genetic classification in clinical use. Therefore, in the present study we aimed to identify simpler and more widely available methods by which gliomas could be diagnosed at many clinical institutions. We focused on Sanger sequencing to address this problem and selected *IDH1/2* and *TP53* as markers for polymerase chain reaction (PCR) analyses. *IDH2* mutations were first detected in gliomas by Yan et al. [3]; similar to *IDH1* mutations, *IDH2* mutation were associated with a better prognosis, although these mutations occurred at considerable lower frequency. Moreover, *TP53* mutations are often detected in astrocytic tumors [7] and it has been shown that these are mutually exclusive with 1p/19q co-deleted gliomas [8]. Therefore, we hypothesized that most *IDH* mutant gliomas without *TP53* mutations carry 1p/19q co-deletions.

Given the increase in CNAs with tumor regrowth or progression to high grade gliomas, according to our CGH analyses, the identification of common and specific CNAs for each genetic subgroup should facilitate an oncological understanding of gliomas. Although we previously reported that 1p/19q co-deletions and +7q are frequently detected in *IDH* mutant gliomas [5] according to our CGH data, only 68% of *IDH* mutant gliomas harbored 1p/19q co-deletions and/or +7q. Therefore, in this study, we also aimed to identify common CNAs in *IDH* mutant gliomas, particularly those harboring *TP53* mutation.

In the present study, we analyzed *IDH1/2* and *TP53* mutations in adult supratentorial gliomas via direct sequencing and characterized these malignancies using PCR-based genetic subgrouping, achieving greater prognostic accuracy than that achieved with pathological classifications. In addition, we confirmed that most *IDH* mutant gliomas with *TP53* mutations contained at least one of the CNAs +7q, +8q, −9p, and −11p. Because *IDH* mutant gliomas with *TP53* mutations showed high recurrence rates, we suggest that these CNAs are negative prognostic factors for patients with *IDH* mutant gliomas.

## Materials and Methods

### Patients, samples, and DNA preparation

We analyzed 237 adult supratentorial glioma samples that had been surgically resected at Keio University from 1994 until 2004 and at Fujita Health University from 2001 until 2015. The histological diagnoses included (anaplastic) astrocytomas, (anaplastic) oligodendrogliomas, (anaplastic) oligoastrocytomas, and glioblastomas. Some patients underwent ≥2 surgeries during their clinical course, thereby contributing to multiple glioma samples. The samples were evaluated by neuropathologists and were classified according to the WHO criteria. Tumor samples were available as frozen tissues and/or as formalin-fixed paraffin-embedded (FFPE) samples. DNA was extracted from freshly frozen tissue using DNeasy blood and tissue kits (QIAGEN) and from FFPE samples with DNA FFPE tissue kits (QIAGEN) or REPLI-g kits (QIAGEN). DNA quality was assessed via absorptiometric analyses. This study was approved by the Ethics Committee of the Fujita Health University (Approval number: 11–106). Written informed consent was obtained from each patient.

### CGH

The CGH analysis was conducted as described by Hirose et al. [1]. Tumor tissues were removed from FFPE samples according to pathological appearance or MIB-1 density and tumor DNA was amplified via degenerate oligonucleotide-primed PCR (DOP-PCR). DNA from peripheral blood lymphocytes was obtained from healthy donors and was used as a control. DNA from these samples was labeled with biotin–deoxyuridine triphosphate (Roche) after amplification. Subsequently, labeled DNA from tumors and normal tissues was hybridized to normal metaphase spreads. After unhybridized probes were washed away, the spreads were counterstained with 4,6-diamino-2-phenylindole and the fluorescence intensity ratios for each chromosome were assessed using CytoVision software (Applied Imaging).

As described previously, total chromosomal gains and partial gains, such as +7 and +7q, were interpreted as different CNAs [5]; +7 was interpreted as a typical copy number change for *IDH* wild-type gliomas and +7q was often detected in *IDH* mutant gliomas. Because gliomas with and without *IDH* mutations are thought to be evolved through different lineages [5], we assumed that the total and partial chromosomal gains would reflect different processes. However, we considered total loss and partial losses (such as −10 and −10q) to be identical CNAs, although they were frequently detected in *IDH* wild-type gliomas that did not show differences in prognosis or histology. Accordingly, we categorized −10 and −10q as −10q.

### Mutation analysis

Sanger sequencing was used to detect *IDH1*/*2* and *TP53* mutations in the samples. We analyzed the sequence of codon 132 for *IDH1* and codon 172 for *IDH2*. In previous studies, most *TP53* missense mutation hotspots were found in exons 5–8 [9–11], and missense mutations in the DNA-binding domains affected the prognosis of patients with breast carcinoma [12]; therefore, we investigated exons 5–8 in *TP53* mutation analyses. The primers used in our study were selected according to previous studies [10, 13–15], and sequence analyses were conducted using ABI 3100 apparatus (Applied Biosystems).

### Statistical analysis

The primary endpoint in this study was progression-free survival (PFS), which is defined as the period from the date of first surgery until the confirmation of tumor regrowth via magnetic resonance imaging (MRI) or symptomatic deterioration. Prognoses were calculated according to PFS and OS, another important factor with respect to a patient’s prognosis. OS was defined as the period from the date of first surgery until the date of death. Kaplan–Meier curves were generated in cases involving first surgery. Cox log-rank tests were used for group comparison. A multivariate logistic regression analysis was conducted to examine correlation between recurrence within 3 years and clinical factors, histology, or specific CNAs. We selected 3 years as the cut-off for recurrence since the median PFS of *IDH* mutant gliomas ranged from 42 to 51 months according to our patient data and a previous study [16] and excluded cases if follow-up months were less than 36 months. We defined subtotal resection (STR) as a tumor resection volume of >90%.

## Results

### Comparison of the PCR-based genetic classification and histological classification

The histological diagnosis of the 237 adult supratentorial gliomas evaluated in this study included astrocytomas, oligodendrogliomas, oligoastrocytomas, and glioblastomas; *IDH1/2* and *TP53* mutation statuses were determined via direct sequencing (S1 Fig). Among the 113 *IDH* mutant gliomas, 42 harbored *TP53* mutations and 42 did not. The remaining 29 samples were from biopsies or were very old; thus those samples provided DNA of insufficient quantity or quality for analyses. Because *TP53* mutations did not affect the prognosis of *IDH* wild-type gliomas according to our study, we classified gliomas as *IDH* mutant gliomas without *TP53* mutations, *IDH* mutant gliomas with *TP53* mutations, and *IDH* wild-type gliomas.

**Fig 1:**
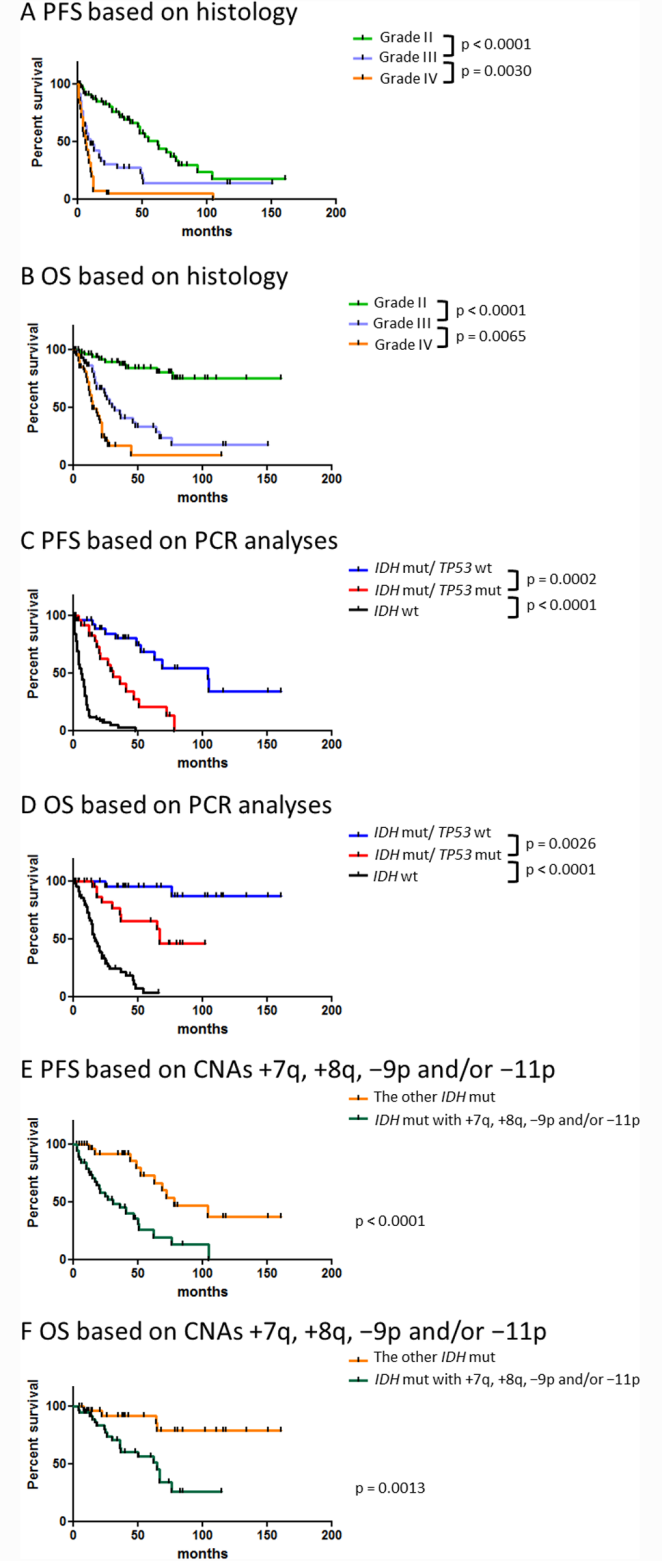
Kaplan–Meier curves of progression-free survival (PFS) according to subgroups. A comparison of PFS and overall survival (OS) according to (A and B, respectively) pathological (n = 171) and (C and D, respectively) genetic classification (n = 158). Kaplan–Meier curves comparing PFS (E) and OS (F) associated with *IDH* mutant gliomas harboring CNAs +7q, +8q, −9p, and/or −11p with the PFS and OS of other *IDH* mutant gliomas (n = 73). Only patients who underwent an initial surgical intervention were included in these analyses. Abbreviations: mut; mutation, wt; wild-type.

Fig 1 shows the prognoses of patients grouped according to histology or genetics. Table present patients background information, including gender, age at diagnosis, recurrence rates, median PFS, and median OS, for each histological and genetic subgroup, respectively. As this was a retrospective study, the study cases had not undergone adjuvant therapy according to strict regimen. Patients with *IDH* wild-type gliomas were significantly older than those with *IDH* mutant gliomas (p < 0.05). Those harboring *IDH* mutant gliomas without *TP53* mutations had a lower recurrence rate relative to the other two subgroups (p < 0.05). Although *IDH* mutant gliomas with *TP53* mutations and *IDH* wild-type gliomas were both associated with high recurrence rates, the median PFS in the latter group was significantly shorter than that in the former group (Table 1B and Fig 1C; hazard ratio = 0.229; 95% confidence interval [CI]: 0.142–0.368; p < 0.0001). In these two genetic subgroups, the median OS of patients with *IDH* wild-type gliomas was also significantly shorter than that of patients with *IDH* and *TP53* mutant gliomas (Table 1B and Fig 1D; hazard ratio = 0.270; 95% CI: 0.155–0.460; p < 0.0001). On the other hand, the median OSs for grade III and IV gliomas were relatively similar (10 and 6 months, respectively) although the difference between grade III and IV gliomas were statistically significant. These results suggests that PCR-based genetic classification provides more precise clinical information, which includes recurrence rates and PFS, than that provided by histological classification.

**Table 1:** Background of patients who underwent initial surgical intervention.

**A**				
		**WHO grade II (n = 63)**	**WHO grade III (n = 48)**	**WHO grade IV (n = 60)**
**Female**		**30**	**20**	**25**
**Age at diagnosis**		**42.6**	**54.0**	**62.4**
***IDH* mutation**		**53**	**30**	**2**
**Prognosis**				
	**Recurrent**	**28**	**33**	**50**
	**Dropped**	**12**	**9**	**7**
	**Following**	**23**	**6**	**3**
**Median PFS (mo)**		**62**	**10**	**6**
**Median OS (mo)**		**Undefined**	**32**	**15**
**B**				
		***IDH* mutant gliomas without *TP53* mutation (n = 32)**	***IDH* mutant gliomas with *TP53* mutation (n = 29)**	***IDH* wild-type gliomas (n = 97)**
**Female**		**14**	**16**	**43**
**Age at diagnosis**		**41.3**	**43.5**	**62.1**
**Histology**				
	**Oligo Grade II**	**19**	**9**	**3**
	**Oligo Grade III**	**8**	**3**	**3**
	**Astro Grade II**	**3**	**9**	**5**
	**Astro Grade III**	**1**	**7**	**14**
	**Astro Grade IV**	**1**	**0**	**55**
	**Not defined/others**	**0**	**1**	**17**
**Additional therapy**				
	**None**	**8**	**10**	**10**
	**Radiotherapy only**	**4**	**2**	**9**
	**Chemotherapy only**	**14**	**5**	**12**
	**Combined**	**5**	**12**	**62**
	**Others or unknown**	**1**	**0**	**4**
**Prognosis**				
	**Recurrent**	**11**	**18**	**81**
	**Dropped**	**3**	**2**	**10**
	**Following**	**18**	**9**	**6**
**Median PFS (mo)**		**104**	**31**	**6**
**Median OS (mo)**		**Undefined**	**67**	**17**

A comparison of patient backgrounds according to histological classification (A) and genetic classification (B). In this table, oligoastrocytomas were classified as oligodendroglial tumor.

### Results of CGH analysis in *IDH* mutant gliomas

**Fig 2:**
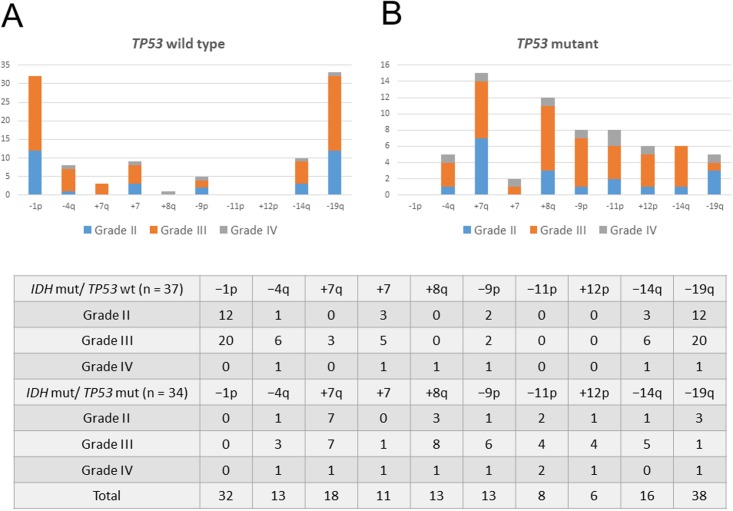
CNAs frequently detected in *IDH* mutant gliomas. A comparison of CNAs found in *IDH* mutant gliomas (A) with wild-type *TP53* and (B) mutant *TP53*. The number of CNAs detected in both *IDH* mutant gliomas with wild-type and mutant *TP53* is summarized in a table.

In a previous study, we reported a high frequency of +7 and −10q CNAs among patients with *IDH1* wild-type gliomas [5]. In this study, we analyzed whole-chromosome gains and losses and identified the CNAs frequently observed in *IDH* mutant gliomas with and without *TP53* mutations (Fig 2). Notably, −1p was uniquely observed in *IDH* mutant gliomas without *TP53* mutations and was always accompanied by −19q. Moreover, the CNAs −4q, +7, −14q, and −19q were mainly detected in *IDH* mutant gliomas without *TP53* mutations. However, +7q and −9p were more frequently found in *IDH* mutant gliomas with *TP53* mutations than in other *IDH* mutant gliomas; +8q, −11p, and +12p were almost exclusively detected in *IDH* mutant gliomas with *TP53* mutations.

### Correlation between *TP53* mutation and CNAs in *IDH* mutant gliomas

**Fig 3:**
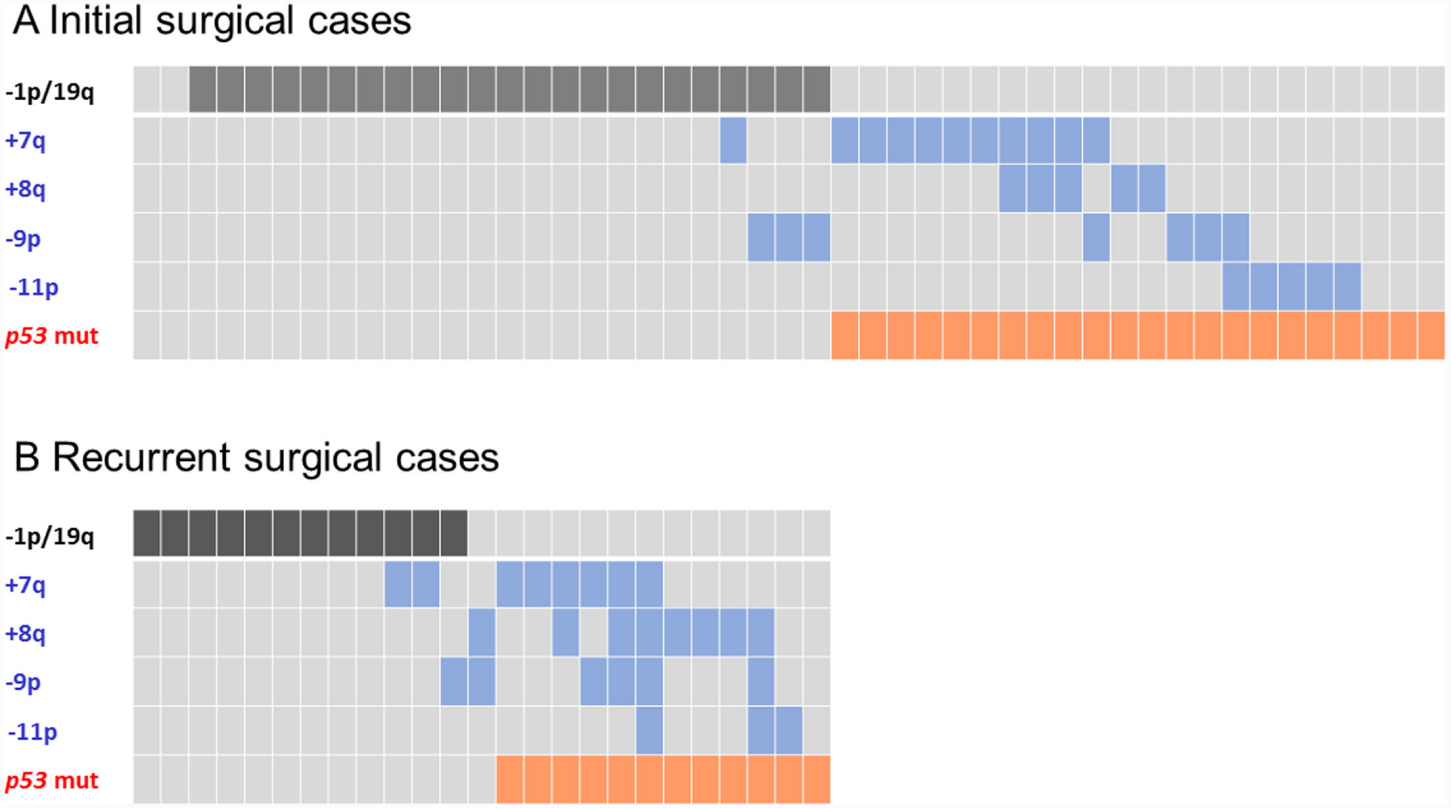
The correlation between *TP53* mutations and the CNAs −1p/19q, +7q, +8q, −9p, and −11p in *IDH* mutant gliomas. A comparison between (A) primary (n = 47) and (B) recurrent disease (n = 24). The CNA −1p/19q represents a favorable prognostic marker; the CNAs +7q, +8q, −9p, and −11p represents unfavorable prognostic markers.

We subsequently investigated the correlation between the *TP53* mutation and CNA statuses. As expected, gliomas with *TP53* mutation and −1p/19q were mutually exclusive. Most *IDH* mutant gliomas with *TP53* mutations had at least one of the CNAs +7q, +8q, −9p, and −11p (Fig 3), and those CNAs overlapped with −1p/19q in tumors lacked *TP53* mutations. *IDH* mutant gliomas with and without +7q, +8q, −9p, and −11p are summarized in Table 2.

**Table 2:** A list of *IDH* mutant glioma patients with (A) and without (B) +7q, +8q, −9p, and/or −11p according to comparative genomic hybridization (CGH) analysis as well as their prognosis and *TP53* mutation status.

**A**									
**Histology**	**Age, Sex**	**Surgery**	**CNAs**	**Genetic type**	***TP53* mut**	**Tumor regrowth**	**PFS, mo**	**Follow-up, mo**	**Outcome**
**A GII**	**22M**	**1** ^**st**^	**+8q21.3-ter, +10pter-q23, −10q25-ter, −X**	**+8q**	**NA**	**No**	**85**	**85**	**Alive**
A GII* ^**1**^	**25M**	**1** ^**st**^	**−4q22-ter, +5pter-23.3, −5q31.2-ter, +7, +8q, +13, −19q, −22**	**+8q**	**NA**	**Yes**	**15**	**25**	**Dead**
**A GII**	**27M**	**1** ^**st**^	**+7q31.3-ter, +10, +20**	**+7q**	**p.K132fs**	**No**	**30**	**30**	**Alive**
**A GII**	**28F**	**1** ^**st**^	**−6q, +8q21.1-ter, −9p, −14q22-ter, −Xp**	**+8q, −9p**	**NA**	**Yes**	**62**	**77**	**Dead**
**A GII**	**30F**	**1** ^**st**^	**−6q, −9p, −14q22-ter, −19q**	**−9p**	**p.D281G**	**Yes**	**41**	**67**	**Alive**
A GII* ^**2**^	**33M**	**1** ^**st**^	**+8q22.3-ter, −12q13-24.1**	**+8q**	**p.R273H**	**Yes**	**12**	**83**	**Alive**
**A GII**	**35F**	**1** ^**st**^	**−4q, +6petr-q21, −8p21, +8q21.1–22, −13q22-ter, −14q21-ter**	**+8q**	**NA**	**Yes**	**76**	**76**	**Alive**
A GII* ^**3**^	**44F**	**1** ^**st**^	**−3p22-21, +7q, +8q22-ter, +11q23.3-ter, +12p, −13q21-31, −19q**	**+7q, +8q**	**p.R273C**	**Yes**	**27**	**65**	**Dead**
**A GII**	**45M**	**1** ^**st**^	**+8q21.1–24.1, −11q23-ter, +19**	**+8q**	**NA**	**No**	**48**	**48**	**Alive**
**A GII**	**46F**	**1** ^**st**^	**+7q, −19q**	**+7q**	**p.H179Y**	**No**	**14**	**14**	**Alive**
**A GII**	**54F**	**1** ^**st**^	**−4q, +8, +10pter-q22.3, −11p**	**−11p**	**p.N247S**	**No**	**15**	**15**	**Alive**
**A GII**	**56F**	**1** ^**st**^	**+7q, −10, +19q, +20**	**+7q**	**NA**	**Yes**	**5**	**9**	**Dead**
**A GII**	**57F**	**1** ^**st**^	**+1q31-ter, −11p, +12p, −12q12-22, +12q23-ter, −18, −19q, −20p, −21**	**−11p**	**NA**	**No**	**2**	**2**	**Alive**
**A GIII**	**27F**	**1** ^**st**^	**−3p21-13, +7p, +8q, +10p, −13q, +14q24.3-ter, −16q, −19q, −X**	**+8q**	**NA**	**Yes**	**13**	**32**	**Dead**
**A GIII**	**35F**	**1** ^**st**^	**−11, −17p, −18q, −20p**	**−11p**	**p.R175H**	**Yes**	**31**	**37**	**Dead**
**A GIII**	**35M**	**1** ^**st**^	**−11, −12q**	**−11p**	**p.R273C**	**No**	**9**	**9**	**Alive**
**A GIII**	**39F**	**1** ^**st**^	**+1q21-31, −1q32.2-ter, +2p16-11.2, −5q, −6q, +7q, +8q23-ter, −9q22-33, −10p, +17q, −19q, −Xp**	**+7q, +8q**	**NA**	**No**	**18**	**18**	**Alive**
**A GIII**	**40F**	**1** ^**st**^	**+10q23-ter, −11, −13, −14, −17p, +17q, −18**	**−11p**	**NA**	**Yes**	**4**	**4**	**Alive**
**A GIII**	**43M**	**1** ^**st**^	**+8q, +12p, -19q, +20, +X**	**+8q**	**NA**	**Yes**	**50**	**50**	**Alive**
**A GIII**	**47F**	**1** ^**st**^	**−8p, −9p, +9q, −10q24-ter, +12q14-15, −12q21-ter, −14q21-31**	**−9p**	**p.R273C**	**Yes**	**21**	**36**	**Dead**
A GIII* ^**4**^	**48M**	**1** ^**st**^	**−4q13-21, +7q, +13q31-ter, +X**	**+7q**	**p.R175H**	**Yes**	**51**	**67**	**Dead**
A GIII* ^**4**^	**52M**	**2** ^**nd**^	**+2pter-22, +3pter-23, +4q21-24, +4q26-33, −6pter-22, −6q21-ter, +7q,−9pter-21, −12p13, +20q**	**+7q, −9p**	**p.R175H**	**Yes**	**1**	**67**	**Dead**
**A GIII**	**55M**	**1** ^**st**^	**−2pter-23, +2p21-13, +3pter-22, +3q24.1-ter, −4q28, +8p, −8q12-13, +8q21.3–22.3, −9p, −10q22.1-ter, +12q13.1–23, −13q12.1–22, −17p, +17q, +19q, −20p**	**+8q, −9p**	**NA**	**Yes**	**3**	**26**	**Dead**
**A GIII**	**59F**	**1** ^**st**^	**−4q, −5p, +7, +8q**	**+8q**	**NA**	**No**	**10**	**10**	**Alive**
**A GIII**	**65F**	**1** ^**st**^	**+1p35-33, +1q, +3p22-21, -6, +7p, +7q32-ter, +8q23-ter, +9pter-21, -9q, −10q, −13, −14, +15q, -22q**	**+7q, +8q**	**p.R244S, p.R245D**	**Yes**	**10**	**30**	**Dead**
A GIII* ^**5**^	**74F**	**2** ^**nd**^	**+1p21, +1q, +7p15-cen, +8q21.3, +10p, +16p11.2, +16q**	**+8q**	**p.A138V**	**No**	**13**	**19**	**Dead**
OA GII* ^**6**^	**34M**	**1** ^**st**^	**−1p, +2p, −9p, −19q**	**−1p/19q, −9p**	**none**	**Yes**	**25**	**34**	**Alive**
OA GII* ^**7**^	**39M**	**1** ^**st**^	**+7q, +10q24-ter**	**+7q**	**p.R175H**	**Yes**	**47**	**60**	**Alive**
OA GII* ^**7**^	**43M**	**2** ^**nd**^	**−5p, +7q, −18q**	**+7q**	**p.R175H**	**No**	**11**	**60**	**Alive**
**OA GII**	**46F**	**1** ^**st**^	**+3p, −5p, +8, −11p, −13q12.1–21.3, +13q22-ter**	**−11p**	**p.R306X**	**No**	**19**	**19**	**Alive**
OA GI * ^**8**^	**61F**	**1** ^**st**^	**+7q31-ter, −X**	**+7q**	**p.Y163C**	**Yes**	**20**	**74**	**Alive**
OA GII* ^**8**^	**63F**	**2** ^**nd**^	**+7q31.1-ter, +12q22-ter, −Xp**	**+7q**	**p.Y163C**	**No**	**7**	**74**	**Alive**
OA GIII* ^**9**^	**30M**	**2** ^**nd**^	**−1p, −4, +7q21.3-ter, +8, +11, −14q22-23, −18, −19q**	**−1p/19q, +7q**	**none**	**Yes**	**16**	**111**	**Alive**
OA GIII* ^**2**^	**34M**	**2** ^**nd**^	**+4p, −4q, −5qcen-13, −5q21-ter, +8q13-ter, −9pter-21.3, −11p, +12p, −12q22-23**	**+8q, −9p, −11p**	**p.R273H**	**Yes**	**48**	**83**	**Alive**
**OA GIII**	**36F**	**1** ^**st**^	**−1q41-ter, −6q, +7q31-ter, −9p, −14q22-ter, −Xq21-ter**	**+7q, −9p**	**p.G245S**	**No**	**36**	**36**	**Alive**
**OA GIII**	**40F**	**1** ^**st**^	**−1p, +1q, +3, −9, +12q14, −15q, +17, +18, −19q, +20**	**−1p/19q, −9p**	**none**	**Yes**	**105**	**115**	**Alive**
OA GIII* ^**10**^	**44F**	**3** ^**rd**^	**+2p, −3p21.3–11.2, +7, +8q23-ter, +10pter-12.3, −19q13.2-ter**	**+8q**	**p.Y220C**	**Yes**	**2**	**80**	**Alive**
OA GIII* ^**3**^	**47F**	**2** ^**nd**^	**−4q28-ter, +7q, +8q23-ter, +12p, −Xq**	**+7q, +8q**	**p.R273C**	**Yes**	**29**	**65**	**Dead**
OA GIII* ^**5**^	**74F**	**1** ^**st**^	**+1p21, +1q, +2p16-ter, +2q, −3p21, +7p21, +7qcen-21, +8, +10p, +10qcen-24, +11, +12p, -12q14-23, +16q**	**+7q**	**p.A138V**	**Yes**	**6**	**19**	**Dead**
**O GII**	**57F**	**1** ^**st**^	**−1p, −9pter-21, −18, −19q, +21**	**−1p/19q, −9p**	**none**	**No**	**51**	**51**	**Alive**
**O GII**	**59M**	**1** ^**st**^	**+7q, +8q21.1-ter, +10p, −13q14-32, −16q, −19q**	**+7q, +8q**	**p.R248W**	**No**	**36**	**36**	**Alive**
**O GII**	**71M**	**1** ^**st**^	**−6pter-16, +8q21.1–21.3**	**+8q**	**NA**	**No**	**40**	**40**	**Alive**
O GIII* ^**11**^	**37F**	**2** ^**nd**^	**−1p, +1q, −2, +6, +7, +8, −9, +11, −16, +17, −18, +19p, −19q, +21, −22**	**−1p/19q, −9p**	**none**	**No**	**8**	**26**	**Alive**
**O GIII**	**41M**	**5** ^**th**^	**−1p, +7q, +8, +18p, −18q, −19q, +22q**	**−1p/19q, +7q**	**none**	**Yes**	**5**	**76**	**Dead**
**O GIII**	**62M**	**1** ^**st**^	**−1p, +7q31.1-ter, −19q**	**−1p/19q, +7q**	**none**	**No**	**0**	**0**	**Dead**
O GIII* ^**12**^	**64M**	**2** ^**nd**^	**−1p, +2, +7, −9p, +9q, −15q, −19q**	**−1p/19q, −9p**	**none**	**Yes**	**27**	**134**	**Alive**
**O GIII**	**72M**	**1** ^**st**^	**+1q, −2q37, −4p, +4q, −6pter-21.3, −6q16-ter, −8, −9pter-23, −11p, −14q, −17p, +17q22-ter**	**−9p, −11p**	**p.R248W**	**Yes**	**4**	**15**	**Dead**
GBM* ^**1**^	**26M**	**2** ^**nd**^	**−3q11.2–24, +3q24.1-ter, +4p, −4q, +5pter-5q23.3, −5q31.2-ter, −6pter-22.1, +6p22.2–18.3, −6q21-26, +7, +8q, −9p, +13, −14q, +16q, +17q, −18, −19q, +21, −22**	**+8q, −9p**	**none**	**Yes**	**1**	**25**	**Dead**
GBM* ^**13**^	**28F**	**2** ^**nd**^	**−3pter-3q24, −5p, +7, −11p, −11q22-23.1, −13q, −19q, −22, −X**	**−11p**	**p.Y220C, p.R248W**	**No**	**24**	**102**	**Alive**
GBM* ^**14**^	**28M**	**3** ^**rd**^	**−4q28-ter, +5pter-q23.3, +7q, +8q, −9p, −9q, −11pter-15.1, −11q23.1-ter, +12p, −13q21.1–22, +13q31-ter**	**+7q, +8q, −9p**	**p.Y236D**	**Yes**	**4**	**67**	**Dead**
**GBM**	**62M**	**1** ^**st**^	**+2, −6p, −7p, +7q, +8q22-ter, −9p, +9q, −11, −13q, −14q, +15q, +18q21**	**+7q, +8q, −9p, −11p**	**NA**	**Yes**	**3**	**4**	**Dead**
ND* ^**14**^	**26M**	**1** ^**st**^	**+7q, +8q22.1-ter, +11q23.3-ter, +12p, +19**	**+7q, +8q**	**NA**	**Yes**	**27**	**67**	**Dead**
ND* ^**14**^	**30M**	**4** ^**th**^	**+7q, +8q, −9p, −X**	**+7q, +8q, −9p**	**p.Y236D**	**Yes**	**4**	**67**	**Dead**
ND* ^**2**^	**38M**	**3** ^**rd**^	**−5q31.1-ter, +8q22.3-ter, +10p, −10q, +12p**	**+8q**	**p.R273H**	**No**	**20**	**83**	**Alive**
**B**									
A GII* ^**13**^	**22F**	**1** ^**st**^	**−11q22-23.1**	**another**	**p.Y220C, p.R248W**	**Yes**	**78**	**102**	**Alive**
A GII* ^**15**^	**22F**	**1** ^**st**^	**none**	**none**	**p.H193Y**	**Yes**	**72**	**85**	**Alive**
**A GII**	**37M**	**1** ^**st**^	**+2q24-33, −3p22-q25, −4q28-ter, +7**	**another**	**NA**	**No**	**7**	**7**	**Alive**
**A GII**	**38F**	**1** ^**st**^	**−1p, −19q**	**−1p/19q**	**none**	**No**	**14**	**14**	**Alive**
**A GII**	**41M**	**1** ^**st**^	**−4**	**another**	**p.H179R**	**No**	**9**	**9**	**Alive**
**A GIII**	**55M**	**1** ^**st**^	**−1p, +7, −10p, −19q**	**−1p/19q**	**NA**	**No**	**12**	**12**	**Alive**
**A GIII**	**60M**	**1** ^**st**^	**−1p, −4, −9q22-ter, −14q21.3-ter, −19q**	**−1p/19q**	**NA**	**No**	**118**	**118**	**Alive**
OA GII* ^**9**^	**24M**	**1** ^**st**^	**−1p, −19q**	**−1p/19q**	**none**	**Yes**	**69**	**111**	**Alive**
**OA GII**	**31F**	**1** ^**st**^	**−12q, −13q14.3–22**	**another**	**none**	**No**	**5**	**5**	**Alive**
**OA GII**	**34F**	**1** ^**st**^	**−1p, −19q**	**−1p/19q**	**none**	**No**	**55**	**55**	**Alive**
**OA GII**	**34M**	**1** ^**st**^	**−1p, −14q, −19q**	**−1p/19q**	**none**	**No**	**161**	**161**	**Alive**
OA GII* ^**16**^	**35F**	**1** ^**st**^	**−1p, −19q**	**−1p/19q**	**none**	**Yes**	**52**	**102**	**Alive**
**OA GII**	**37M**	**1** ^**st**^	**−1p, −14, −19q**	**−1p/19q**	**none**	**Yes**	**35**	**35**	**Alive**
OA GII* ^**10**^	**40F**	**2** ^**nd**^	**+7**	**another**	**p.Y220C**	**Yes**	**22**	**80**	**Alive**
**OA GII**	**41M**	**1** ^**st**^	**−4q26-ter, −5q21-ter, +7, −11q, −12q**	**another**	**none**	**No**	**79**	**79**	**Alive**
**OA GII**	**41F**	**1** ^**st**^	**−1p, −19q**	**−1p/19q**	**none**	**No**	**43**	**43**	**Alive**
**OA GII**	**44F**	**1** ^**st**^	**−1p, −14q, −19q**	**−1p/19q**	**none**	**No**	**81**	**81**	**Alive**
**OA GII**	**45F**	**1** ^**st**^	**−1p, +7, −19q**	**−1p/19q**	**none**	**No**	**5**	**5**	**Alive**
**OA GII**	**48M**	**1** ^**st**^	**−1p, −19q**	**−1p/19q**	**none**	**No**	**39**	**39**	**Alive**
OA GIII* ^**15**^	**28F**	**2** ^**nd**^	**−13q22, +18p, +19, −X**	**another**	**p.H193Y**	**No**	**4**	**85**	**Alive**
OA GIII* ^**17**^	**29M**	**1** ^**st**^	**−1p, −19q**	**−1p/19q**	**none**	**Yes**	**49**	**68**	**Alive**
OA GIII* ^**9**^	**32M**	**3** ^**rd**^	**−1p, +3, −4, +5, +7, +9q, +10p, −10q, −13q, −15q11.2–22.3, +15q22.2-ter, −19q**	**−1p/19q**	**none**	**Yes**	**11**	**111**	**Alive**
OA GIII* ^**17**^	**34M**	**2** ^**nd**^	**−1p, −19q**	**−1p/19q**	**none**	**No**	**9**	**68**	**Alive**
**OA GIII**	**35M**	**1** ^**st**^	**−1p, −4, −13, −18, −19q**	**−1p/19q**	**none**	**No**	**40**	**40**	**Alive**
**OA GIII**	**36M**	**1** ^**st**^	**−1p, −4, +11, −19q**	**−1p/19q**	**none**	**No**	**116**	**116**	**Alive**
**OA GIII**	**37F**	**1** ^**st**^	**−1p, −14q13-24, −19q**	**−1p/19q**	**none**	**No**	**151**	**151**	**Alive**
**OA GIII**	**43M**	**1** ^**st**^	**−1p, +1q12-32.1, −1q32.2-ter, +11, +17, +19p, −19q**	**−1p/19q**	**none**	**Yes**	**44**	**64**	**Dead**
**OA GIII**	**44M**	**1** ^**st**^	**−1p, −19q**	**−1p/19q**	**none**	**No**	**11**	**11**	**Alive**
**OA GIII**	**74M**	**1** ^**st**^	**+1q, +3, +4q12-24, −5q13.1–14, +9p, −14q, −18p**	**another**	**NA**	**No**	**3**	**3**	**Alive**
**O GII**	**33M**	**1** ^**st**^	**−1p, −14q22-24.3, −19q**	**−1p/19q**	**NA**	**No**	**15**	**15**	**Alive**
O GII* ^**18**^	**34M**	**1** ^**st**^	**−1p, −14, −19q**	**−1p/19q**	**none**	**Yes**	**63**	**69**	**Alive**
**O GII**	**52F**	**1** ^**st**^	**−1p, −4, +7, +11q, −15q21-ter, −18q, −19q**	**−1p/19q**	**NA**	**No**	**12**	**12**	**Alive**
O GII* ^**12**^	**53M**	**1** ^**st**^	**−1p, +7, −15q, −19q, +22q**	**−1p/19q**	**none**	**Yes**	**104**	**134**	**Alive**
O GIII* ^**9**^	**34M**	**4** ^**th**^	**−1p, −4, −10q, −13q, −14q, −15qcen-21, +15q24-ter, −19q**	**−1p/19q**	**none**	**Yes**	**9**	**111**	**Alive**
O GIII* ^**11**^	**36F**	**1** ^**st**^	**−1p, −19q**	**−1p/19q**	**none**	**Yes**	**17**	**26**	**Alive**
O GIII* ^**6**^	**36M**	**2** ^**nd**^	**−1p, −17p, −18q, −19q**	**−1p/19q**	**none**	**No**	**7**	**34**	**Alive**
O GIII* ^**18**^	**39M**	**2** ^**nd**^	**−1p, −14q, −19q, +21q**	**−1p/19q**	**none**	**No**	**6**	**69**	**Alive**
O GIII* ^**16**^	**40F**	**2** ^**nd**^	**−1p, +11, −14, −19q**	**−1p/19q**	**none**	**No**	**48**	**102**	**Alive**
O GIII* ^**19**^	**57M**	**2** ^**nd**^	**−1p, −14q13-24, −19q**	**−1p/19q**	**none**	**Yes**	**12**	**85**	**Alive**
O GIII* ^**19**^	**58M**	**3** ^**rd**^	**−1p, +7, −14q21-24.3, −15q15-22.1, −19q**	**−1p/19q**	**none**	**Yes**	**6**	**85**	**Alive**
**O GIII**	**68F**	**1** ^**st**^	**−1p, +2, −3p, +3q, −4, +5, +7, +8, +9q, 13q, +14q, +17, −19q**	**−1p/19q**	**none**	**No**	**21**	**21**	**Alive**

The genetic type indicates detected the CNAs which are regarded as a favorable CNA (–1p/19q) or unfavorable CNAs (+7q, +8q, −9p, and/or −11p). A repeated number denoted by an asterisk indicates ta single patient who underwent multiple surgeries. Abbreviations: NA, not available.

Because *TP53* mutations in *IDH* mutant gliomas were indicative of a poor prognosis and as +7q, +8q, −9p, and −11p were frequently observed in *IDH* mutant gliomas with *TP53* mutations, we hypothesized that these CNAs were associated with a poor prognosis in patients with *IDH* mutant gliomas. Accordingly, patients with *IDH* mutant gliomas who harbored at least one of the abovementioned CNAs had a significantly worse prognosis than did patients with *IDH* mutant gliomas without these CNAs (p < 0.0001; Fig 1E and 1F). The median PFS was 31 months for patients with *IDH* mutant gliomas harboring +7q, +8q, −9p, and/or −11p compared with 78 months for all other *IDH* mutant gliomas (hazard ratio = 0.254; 95% CI: 0.128–0.506; p < 0.0001). The median OS for patients with *IDH* mutant gliomas who harbored these CNAs was 65 months, whereas the median OS for all others could not be defined (hazard ratio = 0.255; 95% CI: 0.111–0.586; p < 0.0001). In addition, a multivariate logistic regression analysis revealed that the 3-year recurrence rate was higher for patients with gliomas who harbored these CNAs than for patients with other types of gliomas (S1 Table). Therefore, +7q, +8q, −9p, and −11p should be considered negative prognostic factors in *IDH* mutant gliomas.

### The CNAs +8q, −9p, −11p, and +12p are candidate markers for tumor progression in *IDH* mutant gliomas

In the present copy number analyses, gliomas with +7q were mainly detected in cases involving first surgeries. However, tumors harboring +8q, −9p, −11p, and +12p were frequently found after subsequent surgeries (Fig 3); +7q, +8q, −9p, −11p, and +12p emerged between the initial surgery and recurrent surgical interventions in 0, 2, 4, 3, and 4 cases of *IDH* mutant gliomas with *TP53* mutations, respectively. Moreover, +7q was frequently detected in grade II gliomas, whereas +8q, −9p, −11p, and +12p were observed in high grade cases. These observations suggest that +7q is an early event, whereas +8q, −9p, −11p, and +12p may reflect tumor progression in *IDH* mutant gliomas with *TP53* mutations. Among patients with *IDH* mutant gliomas with *TP53* mutations, the median PFS was 31 months for gliomas harboring any one of +8q, −9p, −11p, or +12p compared with 47 months for patients harboring all other CNAs (n = 24; p = 0.067). On the other hands, the average MIB-1 indexes was 25.4% among cases harboring +8q, −9p, −11p, and/or +12p and 8.04% in gliomas without these CNAs (n = 31; p = 0.011). These results suggest that malignancy-related genes are present in these regions.

## Discussion

From this study, we report two major findings. First, we have shown that copy number-independent genetic subgroups determined using *IDH1/2* and *TP53* as markers for Sanger sequencing could sufficiently substitute for genetic classification with 1p/19 co-deletions. Second, via a whole-chromosome CNA analysis of *IDH* mutant gliomas with *TP53* mutations, we have clarified the CNAs that contribute to poor prognosis in patients with *IDH* mutant gliomas.

Previous studies have confirmed that specific genetic features including *IDH* mutation and 1p/19p co-deletions are excellent prognostic markers for gliomas [17, 18]. In the present study, we aimed to identify copy number-independent methods that would allow a widespread clinical application of genetic classification of gliomas. Several previous studies reported various prognostic genes identified via mutation analyses, and *ATRX* and *TERT* promoters have recently been recognized as prognostic markers of gliomas [6, 19, 20]. In the present study, we selected *TP53* as a prognostic marker because gliomas with 1p/19q co-deletions and *TP53* mutations were previously shown to be mutually exclusive [8]; accordingly, we hypothesized that *IDH* mutant gliomas with wild-type *TP53* would predominantly harbor 1p/19q co-deletions. Our results support the previous finding that 1p/19q co-deletions and *TP53* mutation are mutually exclusive. The survival curves for patients with gliomas carrying 1p/19q co-deletions were almost identical to those of patients with wild-type *TP53*, suggesting that wild-type *TP53* is sufficiently indicative of 1p/19q co-deletions. In addition to the convenience of PCR-based *TP53* mutation analysis, we are now investigating the relevance of prognosis, CNA, and the mutated *TP53* exon for demonstrating the advantage of subgrouping according to *TP53* mutation versus subgrouping according to 1p/19q co-deletions. *IDH* and *TP53* mutant gliomas that carry +7q also tend to carry mutations in *TP53* exon 5, suggesting that an exon 5 mutation is associated with a better prognosis in *IDH* and *TP53* mutant gliomas comparing with other types of *IDH* and *TP53* mutant gliomas. However, the sample size is extremely low, and we would need to increase the number of analyzed samples to support this conclusion.

As shown in Fig 1E and 1F, *IDH* mutant gliomas harboring any one of the CNAs +7q, +8q, −9p, or −11p were associated with a significantly worse survival when compared with other *IDH* mutant gliomas, indicating that these CNAs are negative prognostic factors for *IDH* mutant gliomas. Several studies previously reported that specific CNAs were candidate negative prognostic markers in gliomas. Our previous studies suggested that gliomas carrying +7q were more likely to be associated with a shorter PFS than were gliomas carrying −1p/19q; −9p was found to be a negative prognostic factor in grade II and III gliomas [1]. Moreover, Kitange et al. and Trost et al. indicated that +8q was associated with short survival durations in patients with oligodendrogliomas [21, 22], and recent studies have reported that −10q, −11p, and −19q were negative prognostic factors for low grade gliomas [16, 23]. Via a whole-chromosome analysis of CNAs for *IDH* mutant gliomas with *TP53* mutations, we clarified that +7q, +8q, −9p, and −11p are unfavorable prognostic factors for *IDH* mutant gliomas. In addition, because +12p was unique to *IDH* mutant gliomas with *TP53* mutations, we suspected that this CNA will be associated with poor survival in patients with *IDH* mutant gliomas. Accordingly, this CNA tended to emerge in cases involving recurrent surgical interventions or high grade gliomas (Figs 2 and 3). However, correlations between gliomas and +12p remain elusive. The chromosomal regions 7q, 8q, 9p, 11p, and 12p contain various oncogenes or tumor suppressor genes, including *MET* (7q31), *MYC* (8q24.21), *CDKN2A* (9p21), *CDKN1C* (11p15.5), and *KRAS* (12p12.1), and these genes might be associated with tumor progression in *IDH* mutant gliomas with *TP53* mutations. The mechanisms underlying the associations of *TP53* mutations with CNAs in the abovementioned specific regions remain unclear. p53 is a transcription factor that regulates target genes in response to DNA damage and is best known as a tumor suppressor gene [24]. Recent studies have correlated the absence of *TP53* with chromosome segregation errors and chromosomal instability [25, 26], suggesting that *TP53* mutations occur during the early phase of tumorigenesis in *IDH* mutant gliomas and cause chromosomal instability and gene dysregulation in specific regions such as 7q, 8q, 9p, 11p, or 12p. Further studies of these chromosomal changes may facilitate interpretations of tumor growth processes in *IDH* mutant gliomas with *TP53* mutations.

Our results confirmed that most *IDH* mutant gliomas with *TP53* mutations involve at least one of the CNAs +7q, +8q, −9p, and −11p, and that most *IDH* mutant gliomas with wild-type *TP53* carry 1p/19q co-deletions. On the other hands, +7 and −10q are frequently detected in *IDH* wild-type gliomas [5]. These results suggest that gliomas can be separated into different lineage depending on *IDH* mutation, and *IDH* mutant gliomas are further separated into two distinct linages according to the *TP53* mutation developing the specific CNAs in each lineage. As mentioned above, *TP53* mutation did not affect the prognosis of patients with *IDH* wild-type gliomas. In a comparison of prognosis between *IDH* wild-type gliomas and primary glioblastomas, the median PFS (6 and 6 months, respectively) and median OS (17 and 15 months, respectively) were almost identical, suggesting that histological diagnosis can sufficiently predict prognosis in cases of primary glioblastomas.

Given the high recurrence rate among *IDH* mutant gliomas with *TP53* mutations, efforts are required to prevent progression to high grade gliomas or secondary glioblastomas, which are difficult to control with multidisciplinary treatments. To this end, studies are in progress now using OncoScan arrays (Affymetrix) for this type of glioma to identify specific regions with common losses, gains, or high copy number gains, and consequent changes in gene expression. In addition, some patients with 1p/19q co-deleted gliomas developed recurrence within a few years and these gliomas lacked *TP53* mutations, suggesting the presence of other genes that contribute to a poor prognoses in patients with *IDH* mutant gliomas.

In this study, we showed that PCR-based mutation analyses using *IDH1/2* and *TP53* as markers could rapidly and simply classify glioma with prognostic relevance. Although pathological diagnoses facilitate evaluations of malignancy at the time of surgery, genetic classifications provide better prognostic predictions, particularly in cases of WHO grade II and III gliomas. Specifically, *IDH* mutant gliomas carrying at least one of the CNAs +7q, +8q, −9p, or −11p were associated with a shorter survival and were predominantly associated with *TP53* mutations. In conclusion, both pathological and genetic classifications are essential for glioma diagnosis and the present observations could be used to facilitate genetic classification.

## Supporting Information

S1 Fig Summary of the histology (A) and *IDH* and *TP53* mutation statuses (B) of participating patients. (TIF)

S1 Table Multivariate analysis of 3-year recurrence among *IDH* mutant gliomas (n = 53). (DOCX)
