# Supplementary figures and images for: PCR-Based Simple Subgrouping Is Validated for Classification of Gliomas and Defines Negative Prognostic Copy Number Aberrations in IDH Mutant Gliomas
Source: PLoS One. 2015 Nov 11;10(11):e0142750. doi: 10.1371/journal.pone.0142750 (PMC4641694; doi:10.1371/journal.pone.0142750)

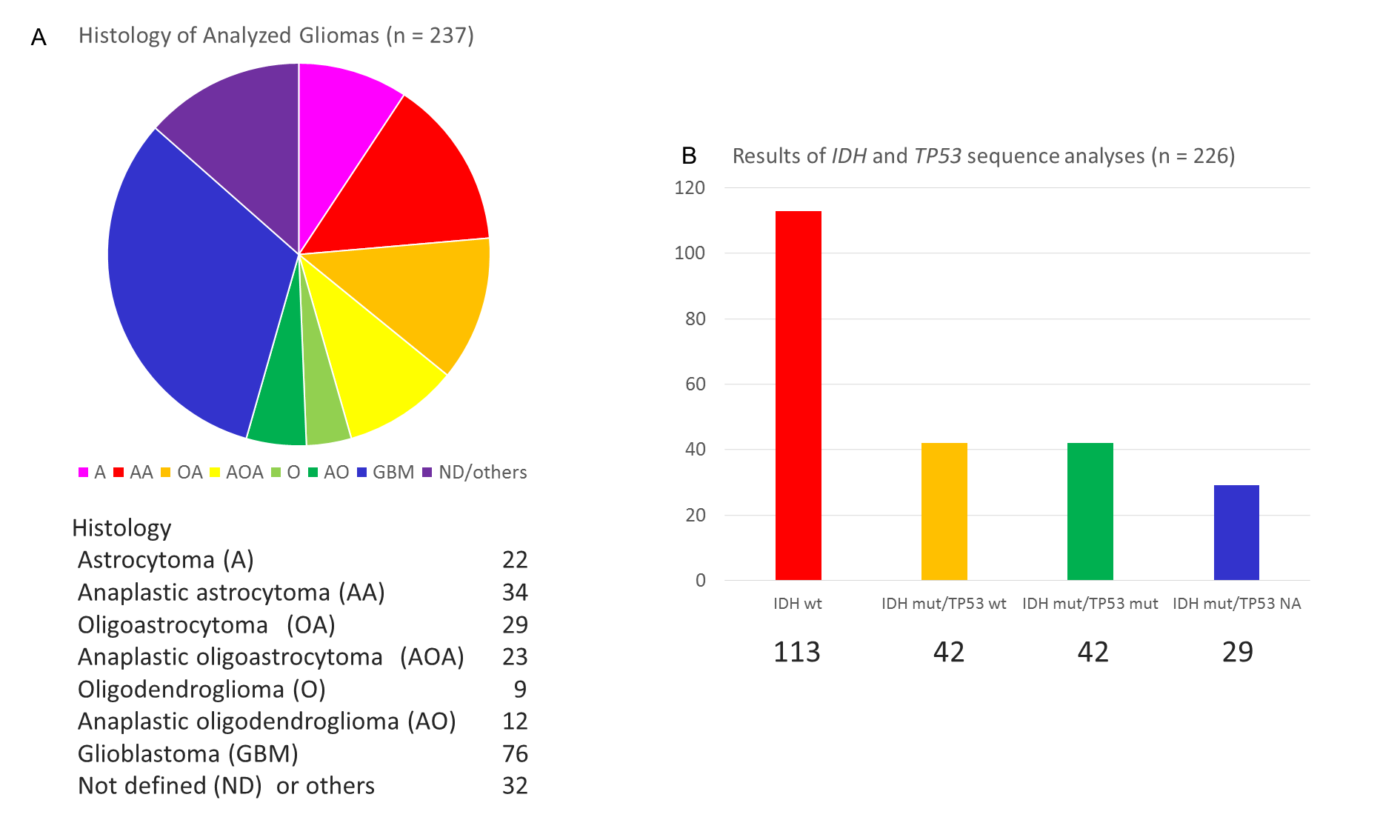

Supplement: S1 Fig — (TIF) [file pone.0142750.s001.tif]
