# Supplementary material for: PCR-Based Simple Subgrouping Is Validated for Classification of Gliomas and Defines Negative Prognostic Copy Number Aberrations in IDH Mutant Gliomas
Source: PLoS One. 2015 Nov 11;10(11):e0142750. doi: 10.1371/journal.pone.0142750 (PMC4641694; doi:10.1371/journal.pone.0142750)
Supplement: S1 Table — (DOCX) [file pone.0142750.s002.docx]

**Supporting Information**

**S1 Fig. Summary of the histology (A) and *IDH* and *TP53* mutation statuses (B) of participating patients.** 237 adult supratentorial gliomas were histologically diagnosed including (anaplastic) astrocytomas, (anaplastic) oligodendrogliomas, (anaplastic) oligoastrocytomas, and glioblastomas. *IDH* and *TP53* mutation statuses were determined via Sanger sequencing.

**S1 Table. Multivariate analysis of 3-year recurrence among *IDH* mutant gliomas (n = 53).** Multivariate analysis of 3-years recurrence after initial surgery was conducted for *IDH* mutant gliomas. The result indicated that partial resection of tumor or biopsy, WHO grade III or IV, and the CNAs +7q, +8q, −9p, and−11p are significantly negative prognostic factors in *IDH* mutant gliomas.

| **Variables** | **OR** | **95% CI** | ***P*-value** |
| --- | --- | --- | --- |
| **Age** | **1.010** | **0.940–1.084** | **0.792** |
| **Gender** | **0.730** | **0.108–4.951** | **0.747** |
| **Surgery: GTR + STR vs. PR + biopsy** | **0.050** | **0.004–0.588** | **0.017** |
| **Chemotherapy vs. none** | **0.936** | **0.093–9.455** | **0.955** |
| **Radiotherapy vs. none** | **9.885** | **0.628–155.704** | **0.103** |
| **Grade II vs. Grade III or IV** | **0.045** | **0.003–0.632** | **0.021** |
| **+7q, +8q, −9p, −11p vs. the others** | **0.013** | **0.001–0.276** | **0.005** |
